# The Functions of ZIP8, ZIP14, and ZnT10 in the Regulation of Systemic Manganese Homeostasis

**DOI:** 10.3390/ijms21093304

**Published:** 2020-05-07

**Authors:** James W.W. Winslow, Kirsten H. Limesand, Ningning Zhao

**Affiliations:** Department of Nutritional Sciences, The University of Arizona, Tucson, AZ 85721, USA; jwwinslow@email.arizona.edu (J.W.W.W.); limesank@email.arizona.edu (K.H.L.)

**Keywords:** manganese, ZIP8, ZIP14, ZnT10, metabolism, metal transporters

## Abstract

As an essential nutrient, manganese is required for the regulation of numerous cellular processes, including cell growth, neuronal health, immune cell function, and antioxidant defense. However, excess manganese in the body is toxic and produces symptoms of neurological and behavioral defects, clinically known as manganism. Therefore, manganese balance needs to be tightly controlled. In the past eight years, mutations of genes encoding metal transporters *ZIP8* (*SLC39A8*), *ZIP14* (*SLC39A14*), and *ZnT10* (*SLC30A10*) have been identified to cause dysregulated manganese homeostasis in humans, highlighting the critical roles of these genes in manganese metabolism. This review focuses on the most recent advances in the understanding of physiological functions of these three identified manganese transporters and summarizes the molecular mechanisms underlying how the loss of functions in these genes leads to impaired manganese homeostasis and human diseases.

## 1. Introduction

Manganese is an abundant element in the Earth’s crust and is naturally found in a variety of minerals [1]. The most common natural form of manganese is manganese dioxide (MnO_2_), also known as pyrolusite, which contains about 60–63% manganese [2]. As a transition metal, manganese exists in different oxidation states, ranging from Mn^3+^ to Mn^7+^, with Mn^2+^ and Mn^3+^ being the most common forms found in biological species [3]. Manganese is widely used in modern industries, such as the production of steel, battery cathodes, soft ferrites for electronics, fertilizers, water treatment chemicals, colorant for automobile undercoating, bricks, frits, glass, textiles, and tiles [4,5,6].

As an essential nutrient, manganese is required for the function of numerous enzymes, including arginase, glycosyltransferases, manganese superoxide dismutase (MnSOD), phosphoenolpyruvate carboxykinase, prolidase, and pyruvate carboxylase [7,8,9,10]. Through these enzymes, manganese plays an important role in human health. Arginase is a manganese-containing enzyme that mediates the conversion of L-arginine to L-ornithine and urea in the last step of the urea cycle pathway [11,12]. The urea cycle is required for protection against the accumulation of excess ammonia. Glycosyltransferases require manganese as a cofactor and are involved in the synthesis of proteoglycans, including mucopolysaccharides, which are necessary for the production of cartilage and bone [13]. MnSOD is a manganese-dependent metalloenzyme that is located in the mitochondria of cells. The primary function of MnSOD is to facilitate the conversion of superoxide radicals to hydrogen peroxide, which is a vital antioxidant defense for nearly all cells [14]. Prolidase is a manganese-activated enzyme that recycles the amino acid proline for collagen synthesis and cell growth [15]. Collagen assembly is required in the process of wound healing. Phosphoenolpyruvate carboxykinase is vital in carbohydrate synthesis from pyruvate and plays an essential role in gluconeogenesis [16].

Deficiency in manganese has been associated with a number of health consequences, including impaired cognitive function, asthma, osteoporosis, and dyslipidemia. Since manganese is present in a broad range of foods and sufficient manganese can be obtained through diet under normal conditions, reports of dietary manganese deficiency are rare [17]. Manganese toxicity, on the other hand, has been observed in individuals drinking contaminated water, in drug addicts who use manganese-contaminated methcathinone, and in children or adults receiving prolonged parenteral nutrition [18]. Moreover, excessive occupational exposure may also occur, which can lead to manganese overload with severe side effects, clinically known as manganism. Individuals who have an increased risk of being exposed to higher than normal levels of manganese include workers in the production of iron and steel, dry cell batteries, fuel oil additives and antiknock agents, glasses, potassium permanganate, fungicides, and leather tanning [19,20,21]. The neurological symptoms of manganism consist of reduced response speed, irritability, intellectual deficits, mood changes, compulsive behaviors in the initial stages of the disorder, and more prominent and irreversible extrapyramidal dysfunction resembling Parkinson’s disease upon protracted exposure [22,23]. Classic clinical features include a mask-like face, limb rigidity, mild tremors, gait disturbance, cock-like walk, slurred speed, excessive salivation, sweating, and disruption in balance [24,25].

**Figure 1:**
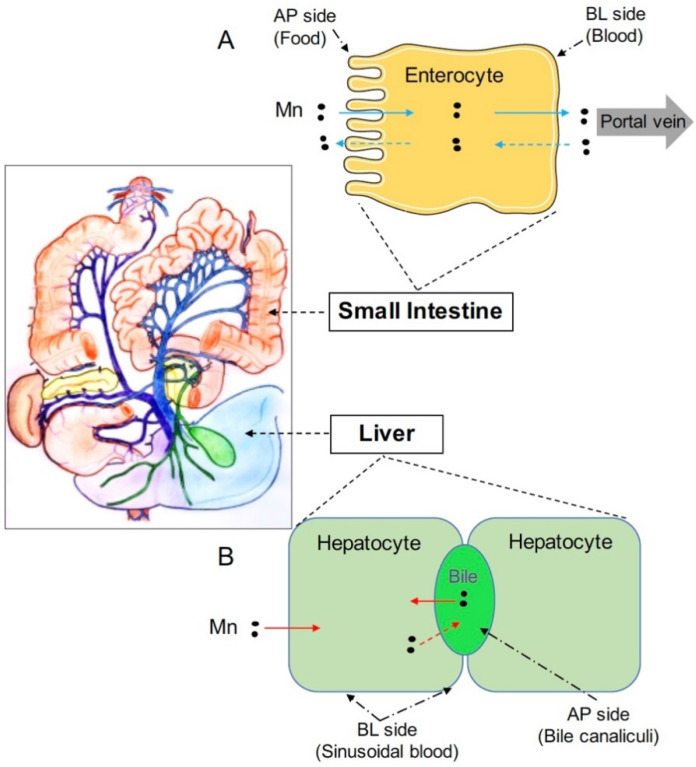
Both the intestine and liver play important roles in regulating systemic manganese metabolism. (**A**) Enterocytes are absorptive cells lining the lumen of intestine. Digested manganese (Mn) in the intestinal lumen can be transported across the apical (AP) membrane into enterocytes and exported across the basolateral (BL) membrane into the blood (solid blue arrows). Absorbed Mn will then be delivered to the liver through the portal vein. Mn in the blood can also be transported into enterocytes and exported back into the intestinal lumen (dashed blue arrows); (**B**) The liver clears Mn from the blood and secretes Mn into the bile for intestinal reabsorption or fecal excretion. Hepatocytes are polarized and represent the major cell type in the liver. The basal domain of hepatocytes faces the sinusoid blood; whereas, the apical domain forms the bile canalicular network. Hepatocytes express polarized, specific transporters that mediate manganese fluxes into or out of these cells (solid red arrows: manganese transport into hepatocytes from the blood or the bile; dashed red arrow: manganese export from hepatocytes into the bile).

Tight homeostatic control is required to meet the dual challenge of avoiding manganese deficiency and preventing manganese overload. At the systemic level, this control is maintained mainly by the intestine and the liver [26,27,28,29]. The intestine regulates dietary manganese absorption; whereas, the liver clears manganese from the blood and secretes this metal as a bile conjugate for subsequent intestinal reabsorption or fecal excretion (Figure 1). At the cellular level, metal transporters responsible for manganese delivery into or out of the cells play important roles in this control. Within the past eight years, mutations of genes encoding metal transporters *ZIP8* (*SLC39A8*), *ZIP14* (*SLC39A14*), and *ZnT10* (*SLC30A10*) have been reported to cause dysregulated manganese homeostasis [18,30,31,32,33], demonstrating that the primary physiological function of these metal transporters is to regulate body manganese metabolism. Among these three proteins, ZIP8 and ZIP14 are members of the Zrt- and Irt-like family proteins (ZIP), and ZnT10 is a member of the Zinc transporter (ZnT) family proteins. In general, the ZIP family proteins control the influx of metals from extracellular fluid or intracellular vesicles into the cytoplasm of the cell; whereas, the ZnT proteins are responsible for the efflux of metals from the cytosol into the extracellular fluid or intracellular vesicles [34]. In this review, we focus on the latest advances in the understanding of the physiological functions of these three manganese transporters and discuss the molecular mechanisms underlying dysregulated systemic manganese homeostasis caused by the deficiency of these genes.

## 2. ZIP8

### 2.1. ZIP8 Mutations and Manganese Deficiency

ZIP8 (also known as SLC39A8, solute carrier 39 family, member 8) is a member of the ZIP metal-ion transporter proteins. The ZIP family takes the name from zinc-regulated transporter (ZRT), iron-regulated transporter (IRT)-like proteins. The *ZIP8* gene encodes a multi-transmembrane protein capable of transporting several divalent cations, including cadmium, zinc, iron, and manganese [35,36,37]. The identification of human diseases associated with *ZIP8* mutations promoted the understanding of the physiological function of this transporter. In 2015, two clinical studies identified *ZIP8* mutations in humans [30,31]. One study described recurrent homozygous *ZIP8* mutations that led to manganese deficiency in a group of children [30]. The analysis of metal levels revealed non-detectable to extremely low levels of blood manganese in affected individuals. The clinical phenotypes of these children include intellectual disability, developmental delay, hypotonia, strabismus, cerebellar atrophy, and variable short stature. Another study identified compound heterozygous *ZIP8* mutations in two unrelated patients with a congenital disorder of glycosylation (CDG) [31]. In this second study, manganese was not detectable in either the blood or urine samples from both patients. Because manganese is essential for glycosyltransferases, glycosylation profiles from both patients confirmed a severe defect in the glycosylation of transferrin, which was used as a marker for plasma proteins. Similar to the first report, clinical phenotypes of both patients include deformities in the skull, severe seizures, short limbs, psychomotor retardation, and hearing loss. In 2017, a homozygous point mutation of *ZIP8* was identified in two siblings to cause severe manganese deficiency, presenting as CDG and Leigh-like syndrome, with standard features of manganese deficiency that included developmental delay, brain atrophy, hypotonia, and seizures [38].

So far, a majority of the identified *ZIP8* mutations stem from consanguineous families, with both parents being heterozygous carriers of the same mutation as that identified in their children. The disease onset occurs at birth or very early during childhood. The detailed information about identified cases of human *ZIP8* mutations are listed in Table 1.

### 2.2. The Molecular Mechanism Underlying ZIP8 Loss and Manganese Deficiency

ZIP8 is ubiquitously expressed throughout the body [39,40,41,42] and detected in almost all cell types with localization to the apical membrane of kidney epithelial cells [43], pulmonary alveolar epithelial cells [44,45], and hepatocytes [46]. In mouse fetal fibroblast cells (MFF cells), retrovirus-introduced ZIP8 functions maximally at pH 7.5 and transports both manganese and cadmium [37]. Overexpression of ZIP8 in HEK293 cells enhanced the cellular uptake of both iron and zinc from the culture media by 200% and 40%, respectively [47]. In *Xenopus* oocytes injected with ZIP8 cRNA, manganese serves as a strong inhibitor for Zn uptake, suggesting a primary role for ZIP8 in mediating manganese uptake into those cells [48]. These in vitro studies have suggested that ZIP8 can import a broad range of metals, including, zinc, iron, cadmium, and manganese.

Conventional whole-body knockout of *Zip8* is embryonic lethal in mice [49]; therefore, to further examine ZIP8’s physiological function, tamoxifen-inducible global *Zip8* knockout (*Zip8*-iKO) mice were generated, and metal concentrations in different organs of these mice were compared to that of wild-type (WT) animals. *Zip8*-iKO mice had markedly reduced manganese in multiple organs, including the liver, brain, kidney, and heart, and presented with decreased manganese-dependent arginase and β-1,4-galactosyltransferase activities [50], which is consistent with the observation that patients carrying *ZIP8* mutations develop severe systemic manganese deficiency. Despite the ability of ZIP8 to transport iron and zinc in cell culture studies, the levels of tissue iron and zinc were not different in *Zip8*-iKO mice compared with that of the control animals, proving that the primary function of ZIP8 is to regulate manganese metabolism. Immunofluorescence analyses determined that ZIP8 is localized to the hepatocyte apical canalicular membrane, suggesting that hepatic ZIP8 may function to mediate manganese transport from the bile into hepatocytes.

**Figure 2:**
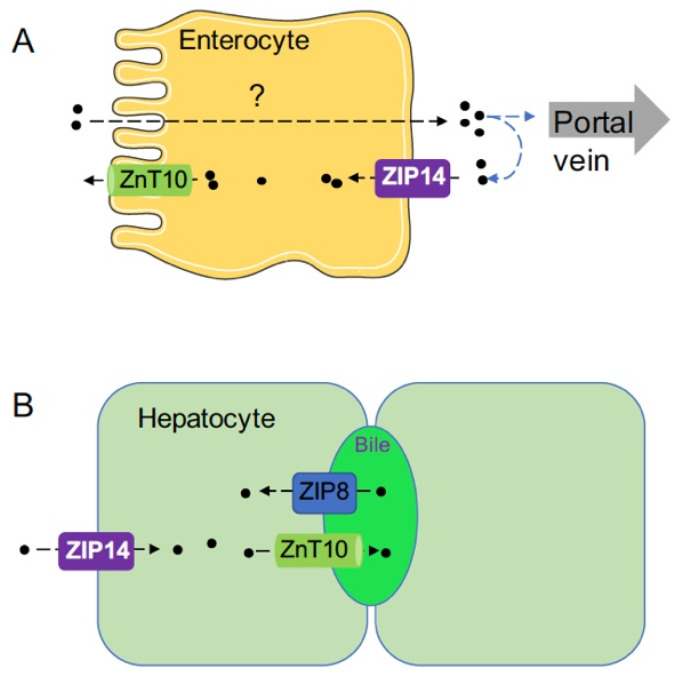
Schematic diagram illustrating the functions of ZIP8, ZIP14, and ZnT10 in enterocytes and hepatocytes. (**A**) The functions of ZIP14 and ZnT10 in enterocytes. How manganese is absorbed from the apical to the basolateral side is not well understood. ZIP14 plays a role in mediating the re-uptake of freshly released manganese at the basolateral membrane. After being reclaimed by enterocytes, manganese can then be transported back into the intestinal lumen by ZnT10 or remains in the intestine epithelium until it is sloughed. Therefore, loss of ZIP14 will decrease manganese re-uptake and increase the net manganese absorption to induce manganese overload. (**B**) The functions of ZIP14, ZnT10, and ZIP8 in hepatocytes. ZIP14 imports circulating manganese into hepatocytes at the basolateral membrane, while ZnT10 exports manganese into the bile at the apical canalicular membrane. ZIP8 reclaims manganese from the bile to increase hepatic manganese storage.

To determine the significance of hepatic ZIP8 in the regulation of whole-body manganese homeostasis, hepatocyte-specific *Zip8* knockout (*Zip8*-L-KO) mice were created [50]. Similar to *Zip8*-iKO mice, *Zip8*-L-KO mice developed manganese deficiency in the liver, brain, kidney, and heart, suggesting that hepatic ZIP8 is required to regulate whole-body manganese homeostasis. Despite reduced whole-body manganese, *Zip8*-L-KO mice had a 55% increased level of manganese in the bile, supporting the role for ZIP8 in mediating transport of manganese from the bile into hepatocytes. Furthermore, liver-specific overexpression of ZIP8 through adeno-associated virus (AAV)-mediated gene delivery into WT mice resulted in a 76% decrease of bile manganese, an 87% increase of liver manganese, and a 94% increase of whole blood manganese, corroborating the view that higher than normal levels of ZIP8 will increase manganese accumulation and possibly lead to manganese overload, presumably through increased manganese reabsorption from the bile [50]. Taken together, these results from genetically modified mouse models demonstrate that ZIP8 localizes to the canalicular domain of hepatocytes to mediate transport of manganese from the bile into hepatocytes (Figure 2B). Thus, ZIP8 may allow reuptake of manganese from the bile when the liver senses deficiencies in manganese, providing one mechanism of manganese deficiency induced by the lack of *ZIP8*. Studies to examine ZIP8 expression under manganese deficiency and overload conditions are needed to further elucidate ZIP8’s function in regulating manganese homeostasis.

## 3. ZIP14

### 3.1. ZIP14 Mutations and Manganese Overload

ZIP14 (SLC39A14) was first identified as a gene responsible for cellular zinc influx based on the observation that overexpression of ZIP14 in Chinese hamster ovary (CHO) cells stimulates the uptake of zinc [51]. Later studies using ZIP14 overexpression models in HEK293 cells, Sf9 insect cells, and *Xenopus* oocytes determined that ZIP14 can mediate the cellular influx of a broad scope of metals, including cadmium, zinc, iron and manganese [52,53,54]. Northern blot analysis detected ubiquitous expressions of ZIP14, with the highest expression in the liver [55]. Human multiple tissue expression array analysis showed that the tissues with high levels of *ZIP14* include the liver and small intestine [51]. A recent genome-wide transcriptomics analysis by RNA-sequencing of human samples from healthy individuals revealed that the greatest ZIP14 abundance was in the liver, followed by the small intestine [41]. In mice, under normal environmental and dietary conditions, the highest expression of ZIP14 was found in the small intestine, followed by the liver [56,57].

The physiological function of ZIP14 became clear with the identification of human mutations. Patients carrying *ZIP14* mutations developed manganese toxicity and early-onset dystonia [32,58,59,60,61]. These patients did not accumulate manganese in the liver and had normal liver function. The levels of other essential metals, including iron and zinc, appeared to be within the normal range, suggesting that the primary physiological role of ZIP14 is to regulate manganese homeostasis. Like patients with *ZIP8* mutations, a majority of *ZIP14* mutation cases stem from consanguineous families. The onset of the disease occurs at infancy or during early childhood and are evident with loss of developmental milestones, progressive dystonia, bulbar dysfunction, spasticity, limb contractures, scoliosis, and loss of independent ambulation, with some showing parkinsonian features of hypomimia, tremor, and bradykinesia. The detailed information about identified human *ZIP14* mutations are listed in Table 2.

### 3.2. The Molecular Mechanism Underlying ZIP14 Deficiency and Manganese Toxicity

Significant insights regarding the disease mechanisms underlying manganese toxicity induced by ZIP14 loss have been gained from animal models with ZIP14 deficiency. ZIP14-deficient zebrafish hyper-accumulated manganese in the brain, but not in the liver, and presented with reduced locomotor activities [32]; whole-body *Zip14* knockout mice (*Zip14*^−/−^) had markedly increased manganese levels in the blood and brain [62,63,64,65], resulting in impaired locomotor behavior, but had decreased liver manganese [62,64,65]. These results clearly demonstrate an indispensable role for ZIP14 in controlling systemic manganese homeostasis and suggest a model where lack of ZIP14 impairs manganese delivery to the liver and subsequent clearance through biliary excretion, which in turn lead to manganese accumulation in the blood and extrahepatic tissues, including the brain [32,62,63,65].

Under normal dietary conditions, hepatocyte-specific *Zip14* knockout mice (*Zip14*-L-KO) had significantly decreased manganese in the liver, confirming the essential function for ZIP14 to import manganese to hepatocytes; however, even with reduced manganese uptake into the liver, *Zip14*-L-KO mice had normal manganese levels in the blood and other tissues [63,66]. These results indicate that although hepatic ZIP14 is required for manganese delivery to the liver, the impaired hepatobiliary manganese excretion alone does not induce manganese hyper-accumulation in individuals lacking functional ZIP14, and suggest that ZIP14 in non-hepatic tissues acts as the primary control for systemic manganese homeostasis during normal physiological situations [63,66].

In addition to the liver, ZIP14 is highly expressed in the small intestine. To examine ZIP14’s function in enterocytes, ZIP14 knockout Caco-2 cells were created [66]. Caco-2 cells have many properties of absorptive enterocytes and have been widely used as a model to examine nutrient transport across the intestinal epithelium. Experiments using the Caco-2 Transwell system have indicated that ZIP14-deletion significantly increased apical-to-basolateral manganese transport. Mechanistically, it has been demonstrated that ZIP14 functions at the basolateral membrane of enterocytes to mediate the re-uptake of freshly released manganese, suggesting a role for ZIP14 in limiting manganese absorption and that a lack-of-ZIP14 in the intestine will increase the net manganese absorption to induce manganese overload (Figure 2A). To determine the physiological relevance of these findings, intestine-specific *Zip14* knockout mice (*Zip14*-In-KO) were generated. In contrast to *Zip14*-L-KO mice that did not develop manganese overload in the body, *Zip14*-In-KO mice developed increased manganese in both the liver and brain under normal dietary conditions, verifying the importance of intestinal ZIP14 in maintaining systemic manganese homeostasis [66,67]. Based on the results from these most recent studies, we propose a new model for the function of ZIP14 in regulating systemic manganese homeostasis that includes the functions of intestinal ZIP14 to limit dietary manganese absorption and hepatic ZIP14 to clear manganese from the portal blood (Figure 2A,B). Future studies are needed to elucidate ZIP14’s role in other organs.

## 4. ZnT10

### 4.1. ZnT10 Mutations and Manganese Overload

ZnT10 (also known as SLC30A10, solute carrier 30 family, member 10) is a member of the Zinc transporter (ZnT) family proteins. In contrast to the function of ZIP members that increase cytosolic metal levels, ZnT proteins function to efflux metals from the cytoplasm either out of the cell or into intracellular organelles [68,69]. As the last member of the ZnT family, ZnT10 was identified by searching the human genome for homologous sequences of known ZnT proteins [70]. The amino acid sequence of ZnT10 was similar to that of ZnT1, which has been shown to principally mediate cellular zinc efflux [71,72]. Gene expression profiles indicated that ZnT10 was highly expressed in the small intestine and liver in both humans and mice [41,42,73]. In neuronal SH-SY5Y cells, Interleukin-6 treatment decreased ZnT10 expression and reduced manganese efflux [74]. In HeLa cells, ZnT10 was detected on the cell surface; and ZnT10 overexpression protected cells from manganese-induced cell toxicity by reducing intracellular manganese [75]. In lymphocyte-derived DT-40 cells, treated with high levels of zinc or manganese, ZnT10 had no effect on zinc export, but was determined to be the primary transporter mediating manganese efflux [76]. These cell culture studies suggest that ZnT10 functions as a manganese exporter to prevent manganese toxicity, which reflects the clinical status of patients carrying *ZnT10* mutations.

In 2008, a clinical case report first described a 12-year old girl with severe dystonia and significantly elevated blood manganese [77]. No history of manganese exposure was noted, and her plasma levels of copper and zinc were within the normal range. This girl was born to healthy consanguineous parents. Her older brother died at 18 years of age and presented with the same disease phenotype prior to death, suggesting a genetic disorder of autosomal recessive inheritance within the family. However, the primary genetic variant and the pathophysiology of this disorder was not revealed at that time. The gene responsible for this disorder was later identified to be *ZnT10* when two additional patients, presented with a similar clinical phenotype, were reported in 2012 [18]. At the same time, another research group also reported the identification of *ZnT10* mutations as the causal gene defect in two consanguineous families with neurological disorders and juvenile-onset severe manganese overload [33]. Individuals with *ZnT10* mutations are evident with phenotypical features of hypermanganesemia with dystonia. Genetic evaluations revealed that in the majority of identified cases, both parents of the affected individuals were heterozygous carriers of the same mutations found in their children. Neurological symptoms can occur during childhood (early-onset) or adulthood (adult-onset). In early-onset cases, children will experience symptoms that include dystonia in the arms or legs, involuntary trembling, unusually slow movement, and slurred speech. In adult-onset, symptoms include movement abnormalities, tremor, extraordinarily slow motion, muscle rigidity, and postural instability [33]. Additional symptoms may consist of polycythemia, low levels of iron in the body, enlarged liver from manganese accumulation, scarring in the liver, and irreversible liver cirrhosis [78,79,80]. Details about identified human *ZnT10* mutations are listed in Table 3.

### 4.2. The Molecular Mechanism Underlying ZnT10’s Function in Manganese Metabolism

The identification of human *ZnT10* mutations indicated that ZnT10 plays a pivotal role in regulating manganese homeostasis. While blood manganese concentrations are notably higher in individuals carrying homozygous *ZnT10* mutations, zinc and iron concentrations in the blood either remain normal or show subtle changes compared to the standard values [18,33,81], further emphasizing the function of ZnT10 in manganese metabolism. Moreover, individuals affected by *ZnT10* mutations also present with high levels of hepatic manganese [18]. Consistent with the human phenotype, ZnT10-deficient zebrafish had significantly increased body manganese, while levels of iron and zinc remained similar to the controls [84]; mice with constitutive whole-body *Znt10* knockout (*Znt10*^−/−^ mice) developed markedly elevated manganese in the liver, brain, and blood, with no changes or minor changes in body iron and zinc contents [64,85,86].

Because immunohistochemistry analysis of human liver tissues found that ZnT10 was abundant in hepatocytes and localized both intracellularly and on the plasma membrane facing the bile duct, it was initially postulated that the primary function of ZnT10 is to remove manganese from hepatocytes for biliary excretion and that the systemic manganese hyperaccumulation observed in individuals lacking ZnT10 is secondary to the defect of hepatic ZnT10 [33]. To determine ZnT10’s function in the liver, hepatocyte-specific *Znt10* knockout mice (*Znt10*-L-KO) were generated [86,87]. After intra-orbital injection of manganese, *Znt10*-L-KO mice had almost no manganese excretion through the bile, confirming that ZnT10 is essential for biliary manganese excretion [86] (Figure 2B). However, in contrast to *Znt10*^−/−^ mice that had 20–60 times more manganese in different tissues of the body, *Znt10*-L-KO mice only developed minimal excess of tissue manganese (about 2-fold increase compared to controls), indicating that ZnT10 in other organs may compensate for the loss of hepatic ZnT10 [86,87].

Besides the liver, the intestine also expresses high levels of ZnT10. To investigate the function of ZnT10 in enterocytes, polarized Caco-2 cells grown on Transwell inserts were used as a model to examine manganese transport. Immunofluorescence analysis and manganese transport assay indicated that ZnT10 localizes to the apical domain of Caco-2 cells and mediates the efflux of intracellular manganese at the apical membrane [87]. This function of ZnT10 may provide a compensatory mechanism to remove manganese into the intestinal lumen when hepatic *ZnT10* is not present. To test the physiological significance of these findings, intestine-specific *Znt10* knockout mice (*Znt10*-In-KO) and mice with *Znt10* deletion in both hepatocytes and enterocytes (*Znt10* double knockout (*Znt10*-DKO)) were created [86]. Similar to *Znt10*-L-KO mice, *Znt10*-In-KO mice only had moderate manganese accumulation in the body, despite reduced manganese export into the intestinal lumen. With a combined deletion of ZnT10 in hepatocytes and enterocytes, *Znt10*-DKO mice developed manganese overload that was more severe than that seen in *Znt10*-L-KO mice or *Znt10*-In-KO mice, but less severe than the manganese loading observed in the whole-body *Znt10*^−/−^ mice. Together, these results demonstrated that hepatic and intestinal ZnT10 both contribute to the regulation of manganese homeostasis and that in addition to the liver and intestine, other organs with ZnT10 expression also contribute to the regulation of systemic manganese homeostasis. Future studies are required to determine ZnT10’s function at other sites.

## 5. Conclusions and Perspectives

Maintaining manganese balance within the body is essential for health. The discoveries of ZIP8, ZIP14, and ZnT10 as crucial manganese transporters have opened new paths towards a better understanding of manganese homeostatic regulation. Experiments using genetically modified animal models with global and tissue-specific knockouts of these genes have provided significant insights into the molecular basis of how deficiency in these genes leads to disorders of manganese metabolism.

The systemic manganese homeostasis is regulated mainly by intestinal absorption and hepatobiliary excretion. In enterocytes of the intestine, ZIP14 localizes to the basolateral membrane and imports extracellular manganese from the circulation into enterocytes, while ZnT10 functions at the apical membrane to export intracellular manganese into the lumen of the intestine. These two transporters function in the secretory direction to restrict the amount of manganese being absorbed into the blood (Figure 2A). With intestine-specific knockout of ZIP14 or ZnT10, mice develop manganese overload due to increased manganese absorption, suggesting that intestinal control of manganese absorption serves as a primary regulation for systemic manganese homeostasis. Future studies are required to address mechanisms for dietary manganese uptake at the apical membrane and manganese export at the basolateral membrane.

In hepatocytes of the liver, ZIP14 is expressed at the basolateral membrane to import circulating manganese into hepatocytes, while ZnT10 localizes at the apical canalicular membrane to export manganese into the bile. Both transporters work in the same direction to facilitate manganese removal through biliary excretion (Figure 2B). As a transporter functioning in the opposite direction, ZIP8 reclaims manganese from the bile to increase hepatic manganese storage. Continued studies are needed to explore the functions of these three manganese transporters in other organs to further advance our knowledge of manganese biology.

## Figures and Tables

**Table 1:** Identified human cases of *ZIP8* mutation.

Subject and Reference	*ZIP8* Mutation	Amino Acid Change	Gender	Age of Onset	Blood Manganese
A-1 ƒ § Ç [30]	c.[112G>C];[112G>C]	p.[Gly38Arg]	F	Birth	ND
B-1 ƒ § Ç [30]	c.[112G>C];[112G>C]	p.[Gly38Arg]	M	Birth	20 nmol/L (Erythrocyte) (NR 273–728)
C-1 ƒ § Ç [30]	c.[112G>C];[112G>C]	p.[Gly38Arg]	M	Birth	20 nmol/L (NR 78–289)
D-1 ƒ § Ç [30]	c.[112G>C];[112G>C]	p.[Gly38Arg]	F	Birth	14.2 nmol/L (NR 5.3–40.8)
D-2 ƒ § Ç [30]	c.[112G>C];[112G>C]	p.[Gly38Arg]	F	Birth	5.5 nmol/L (NR 5.3–40.8)
E-1 ƒ § Ç [30]	c.[112G>C];[112G>C]	p.[Gly38Arg]	M	Birth	18.4 nmol/L (NR 5.3–40.8)
F-1 ƒ § Ç [30]	c.[112G>C];[112G>C]	p.[Gly38Arg]	F	Birth	1.1 mcg/L (NR 5–12.4)
F-2 ƒ § Ç [30]	c.[112G>C];[112G>C]	p.[Gly38Arg]	M	Birth	1.1 mcg/L (NR 5–12.4)
F-3 ƒ § Ç [30]	†	†	N/A	Birth	N/A
G-1 £ [31]	c.[112G>C];[1019T>A]	p.[Gly38Arg];[Ile340Asn]	F	<4 months	ND
H-2 £ [31]	c.[97G>A;1004G>C];[610G>T]	p.[Val33Met; Ser335Thr]; [Gly204Cys]	F	<1 year	ND
I-1 § Ç [38]	c.[338G>C];[338G>C]	p.[Cys113Ser]	F	4 months	ND
I-2 § Ç [38]	c.[338G>C];[338G>C]	p.[Cys113Ser]	F	3 months	ND

Individual families are letters A–I with affected sibling listed. Abbreviations and symbols: Not detectable (ND); normal range (NR); male (M); female (F); †: DNA was not available for testing from the third affected member due to death at 7 months. The individual may have been affected by the same disorder and genetic mutation; ƒ: the mutation was present in two different families; £: expressed compound heterozygous mutations; §: both parents are heterozygous carriers of the identified mutation; Ç: patients from consanguineous families.

**Table 2:** Identified human cases of *ZIP14* mutation.

Subject and Reference	*ZIP14* Mutation	Amino Acid Change	Gender	Age of Onset	Blood Manganese
A-1 § Ç [32]	c.[292T>G];[292T>G]	p.[Phe98Val]	F	7 Months	2887 nmol/L (NR 73–325)
A-2 § Ç [32]	c.[292T>G];[292T>G]	p.[Phe98Val]	F	6 Months	N/A
B-1 § Ç [32]	c.[313G>T];[313G>T]	p.[Glu105*]	F	7 months	8101 nmol/L (NR 73–325)
B-2 § Ç [32]	†	†	F	7 months	N/A
C-1 § Ç [32]	c.[477_478del];[477_478del]	p.[S160Cysfs*5]	F	3 years	963 nmol/L (NR 73–325)
D-1 Ç [32]	c.[1147G>A];[1147G>A]	p.[Gly383Arg]	M	10 months	965 nmol/L (NR 145.6)
E-1 § Ç [32]	c.[1407C>G];[1407C>G]	p.[Asn469Lys]	F	2 years	2280 nmol/L (NR 73–325)
E-2 § Ç [32]	c.[1407C>G];[1407C>G]	p.[Asn469Lys]	F	2 years	3830 nmol/L (NR 73–325)
E-3 § Ç [32]	c.[1407C>G];[1407C>G]	p.[Asn469Lys]	M	2 years	1260 nmol/L (NR 73–325)
F-1 § Ç [60]	c.[311G>T];[311G>T]	p.[Ser104Ile]	M	11 months	10.5 mcg/L (Plasma) (NR 0.4–0.9)
F-2 § Ç [60]	‡	‡	M	10 months	N/A
G-1 § [61]	c.[382C>T];[382C>T]	p.[Arg128Trp]	F	2 months	3640 nmol/L (NR 73–375)
H-1 ƒ § Ç [59]	c.[751-9C>G];[751-9C>G]	p.[His251Profs^*^26]	F	8 months	64.2 mcg/L (Serum) (NR 4–16.5)
I-1 ƒ [59]	c.[751-9C>G];[751-9C>G]	p.[His251Profs^*^26]	F	18 months	78 mcg/L (Serum) (NR 4–16.5)
J-1 § Ç [58]	c.[1136C>T];[1136C>T]	p.[Pro379Leu]	F	15 months	150 nmol/L (NR <10)

Individual families are letters A–J with affected sibling listed. Abbreviations and symbols: Not detectable (ND); normal range (NR); male (M); female (F); †: DNA was not available for testing from the third affected member. Her clinical phenotype was similar to her siblings, suggesting that she may have been affected by the same disorder and genetic mutation; ‡: DNA of F-2 was not available for testing from the second affected member. His clinical phenotype was similar to his sibling (F-1), suggesting that he may have been affected by the same disorder and genetic mutation; ƒ: the mutation was present in two different families; §: both parents are heterozygous carriers of the identified mutation; Ç: patients from consanguineous families.

**Table 3:** Identified human cases of *ZnT10* mutation.

Subject and Reference	*ZnT10* Mutation	Amino Acid Change	Gender	Age of Onset	Blood Manganese
A-1 § Ç [18]	Deletion of exons 1 and 2	N/A	F	3 years	6180 nmol/L (NR <320)
A-2 § Ç [18]	Deletion of exons 1 and 2	N/A	F	3 years	3767 nmol/L (NR <320)
A-3 § Ç [18]	Deletion of exons 1 and 2	N/A	M	5 years	5096 nmol/L (NR <320)
A-4 § Ç [18]	Deletion of exons 1 and 2	N/A	M	5 years	6370 nmol/L (NR <320)
B-1 Ç Ð [33]	c.[507delG];[500T>C]	p.[Pro170Leufs*22]	M	2 years	231.6 nmol/L (NR <32.8)
B-2 Ç Ð [33]	c.[507delG];[500T>C]	p.[Pro170Leufs*22]	M	14 years	2626 nmol/L (NR 183–352)
B-3 Ç Ð [33]	†	†	F	10 years	N/A
C-1 Ç Ð [33]	c.[1235delA];[1235delA]	p.[Gln412Argfs*26]	M	47 years	104 mcg/L (NR 3–8)
C-2 Ç Ð [33]	c.[1235delA];[1235delA]	p.[Gln412Argfs*26]	M	57 years	106 mcg/L (NR 3–8)
D-1 § Ç [18]	c.[266T>C];[266T>C]	p.[Leu89Pro]	F	2 years	2109 nmol/L (NR <320)
D-2 § Ç [18]	c.[266T>C];[266T>C]	p.[Leu89Pro]	F	2 years	1636 nmol/L (NR <320)
D-3 § Ç [18]	c.[266T>C];[266T>C]	p.[Leu89Pro]	F	2 years	1600 nmol/L (NR <320)
E-1 § [18]	c.[292_402del];[292_402del]	p.[Val98_Phe134del]	F	4 years	1145 nmol/L (NR <320)
F-1 § Ç [18]	c.[314_322del];[314_322del]	p.[Ala105_Pro107del]	M	2 years	N/A
F-2 § Ç [18]	c.[314_322del];[314_322del]	p.[Ala105_Pro107del]	F	11 years	3285 nmol/L (NR <320)
G-1 § [18]	c.[585del];[585del]	p.[Thr196Profs*17]	M	14 years	3480 nmol/L (NR <320)
H-1 § [18]	c.[765_767del];[765_767del]	p.[Val256del]	F	11 years	3272 nmol/L (NR <320)
I-1 § Ç [18]	c.[922C>T];[922C>T]	p.[Gln308*]	M	2 years	NA
I-2 § Ç [18]	c.[922C>T];[922C>T]	p.[Gln308*]	F	3 years	3114 nmol/L (NR <320)
J-1 § [18]	c.[1046T>C];[1046T>C]	p.[Leu349Pro]	F	5 years	2366 nmol/L (NR <320)
K-1 § Ç [81]	c.[496_553del58];[496_553del58]	p.[Ala166Glnfs*7]	F	3 years	9.8 mcg/L (serum) (NR 0.3–1.8)
L-1 § Ç [81]	c.[492delC];[492delC]	p.[Gly165Alafs*27]	M	3 years	19.5 mcg/L (serum) (NR 0.3–1.8)
L-2 § Ç [81]	c.[492delC];[492delC]	p.[Gly165Alafs*27]	F	1 year	23.9 mcg/L (serum) (NR 0.3–1.8)
L-3 § Ç [81]	c.[492delC];[492delC]	p.[Gly165Alafs*27]	F	3 years	29.5 mcg/L (serum) (NR 0.3–1.8)
M-1 § Ç [81]	c.[460C>T];[460C>T]	p.[Gln154*]	F	4.5 years	42 mcg/L (serum) (NR 0.3–1.8)
N-1 Ç € [80]	c.[1006C>T]	p.[His336Tyr]	M	10 years	14,972 nmol/L (NR <320)
N-2 Ç € [80]	c.[1006C>T]	p.[His336Tyr]	M	8 years	1511 nmol/L (NR <320)
N-3 Ç € [80]	c.[1006C>T]	p.[His336Tyr]	M	6 years	539 nmol/L (NR <320)
O-1 § Ç [79]	c.[359G>A]	p.[Gly120Asp]	M	4 years	2808 nmol/L (NR 100–260)
O-2 § Ç [79]	c.[359G>A]	p.[Gly120Asp]	M	6 years	2056 nmol/L (NR 100–260)
P-1 § Ç [78]	c.[957 + 1G>C] (Splice site mutation)	N/A	M	10 years	2900 nmol/L (NR <320)
P-2 § Ç [78]	c.[957 + 1G>C] (Splice site mutation)	N/A	M	2 years	3340 nmol/L (NR <320)
Q-1 § Ç [78]	c.[119A>C]	p.[Asp40A]	M	1 year 6 months	3200 nmol/L (NR <320)
R-1 § Ç [78]	c.[122_124delCCT]	p.[Ser41del]	M	1 year 6 months	3310 nmol/L (NR <320)
S-1 § Ç [78]	c.[90C>G]	p.[Tyr30*]	M	1 year 3 months	2980 nmol/L (NR <320)
T-1 § Ç [78]	c.[780_782delCAT]	p.[Iso260del]	F	1 year 6 months	3125 nmol/L (NR <320)
T-2 § Ç [78]	c.[780_782delCAT]	p.[Iso260del]	F	1 year 3 months	3300 nmol/L (NR <320)
U-1 ƒ § Ç [78]	c.[77T>C]	p.[Leu26Pro]	M	2 year	3245 nmol/L (NR <320)
U-2 ƒ § Ç [78]	c.[77T>C]	p.[Leu26Pro]	F	1 year 6 months	3120 nmol/L (NR <320)
V-1 ƒ § Ç [78]	c.[77T>C]	p.[Leu26Pro]	F	4 years	2750 nmol/L (NR <320)
W-1 ƒ § Ç [82]	c.[1006C>T]	p.[His336Tyr]	M	10 years	3000 nmol/L (NR <320)
X-1 § Ç [83]	c.[1188dup]	p.[Leu397Thrfs*15]	F	1 year 6 months	1946 nmol/L (at age of 7) (NR <273)

Individual families are letters A–X with affected sibling listed. Abbreviations and symbols: Not detectable (ND); normal range (NR); male (M); female (F);†: DNA was not available for testing from the third affected member. Her clinical phenotype was similar to her siblings, suggesting that she may have been affected by the same disorder and genetic mutation; ƒ: the mutation was present in two different families; §: both parents are heterozygous carriers of the identified mutation; Ç: patients from consanguineous families; Ð: DNA from parents was not available for testing; €: father and one unaffected sibling were heterozygous for the mutation.
